# Effect of Eye-Object Distance on Body Sway during Galvanic Vestibular Stimulation

**DOI:** 10.3390/brainsci8110191

**Published:** 2018-10-23

**Authors:** Osamu Aoki, Yoshitaka Otani, Shinichiro Morishita

**Affiliations:** 1 Faculty of Rehabilitation, Shijonawate Gakuen University, Osaka 5740011, Japan; 2 Institute of Health Sciences, Shijonawate Gakuen University, Osaka 5740011, Japan; 3 Faculty of Rehabilitation, Kobe International University, Hyogo 6580032, Japan; ohtani@kobe-kiu.ac.jp; 4 Institute for Human Movement and Medical Sciences, Niigata University of Health and Welfare, Niigata 9503102, Japan; morishita@nuhw.ac.jp

**Keywords:** galvanic vestibular stimulation, center of pressure, vision, sway, eye-object distance

## Abstract

Gazing at objects at a near distance (small eye-object distance) can reduce body sway. However, whether body sway is regulated by movement in the mediolateral or anteroposterior direction remains unclear. Galvanic vestibular stimulation (GVS) can induce body tilting in the mediolateral or anteroposterior direction. This study examined the directionality of the eye-object distance effect, using body-tilting GVS manipulations. Ten healthy subjects (aged 21.1 ± 0.3 years) stood on a force plate covered with a piece of foamed rubber and either closed their eyes or gazed at a marker located 0.5 m, 1.0 m, or 1.5 m in front of them. The GVS polarities were set to evoke rightward, forward, and backward body tilts. To compare the effects of eye-object distance in the mediolateral and anteroposterior directions, the root mean square (RMS) of the center of pressure (COP) without GVS was subtracted from the COP RMS during GVS. For swaying in the mediolateral direction, significant visual condition-related differences were found during rightward and forward GVS (*p* < 0.05). Thus, reductions in mediolateral body sway are more evident for smaller eye-object distances during rightward GVS. It would be appropriate to use body-tilting GVS to detect the directionality of the eye-object distance effect.

## 1. Introduction

Information from multiple sensory systems (visual, vestibular, and proprioceptive) is integrated in the central nervous system to provide appropriate physical responses and achieve postural control during upright standing [1]. The “sensory reweighting” hypothesis proposes that the central nervous system dynamically adjusts the contribution of different sensory inputs to control posture [2]. For example, vision is thought to make a larger contribution to balance control, compared with other sensory systems. Gazing at nearby objects (small eye-object distance) can reduce body sway during upright standing [3,4], this phenomenon may be caused by the ability to detect vision and eye movement information, such as proprioception of oculomotor muscles and motion parallax [5]. Therefore, small eye-object distances might facilitate the sensory reweighting of visual information during postural responses. However, it remains controversial whether this effect is evident as body sway in both mediolateral (ML) and anteroposterior (AP) directions [3,6], or only in the ML direction [7,8].

Galvanic vestibular stimulation (GVS) can induce body tilting in a specific (ML or AP) direction, depending on electrode placement and direct current (DC) polarity [9], resulting in deviations and fluctuations in the center of pressure (COP). Furthermore, sensory reweighting of visual information may occur during GVS, as visual feedback from body mass movements has been effective in decreasing the ML COP sway power during sinus wave GVS-induced body-tilting [10].

Although eye-object distance does not provide direct feedback on body mass movements, it may provoke sensory reweighting by providing information on body sway, for example, via oculomotor muscle proprioception and motion parallax. We postulated that the information received from GVS-induced body tilt in the ML and AP directions differs, because the vergence caused by AP body sway [4] would result in less retinal slip and changes in proprioception [7]. Thus, ML and AP body-tilting manipulations might be useful for investigating the directional effectiveness of eye-object distance. Therefore, the purpose of this study was to investigate the extent to which body sway was suppressed by small eye-object distances during body tilting in both directions, using GVS manipulations with healthy subjects.

## 2. Methods

### 2.1. Subjects

Ten healthy subjects with no history of visual or vestibular disease (three men and seven women; age (mean ± SD): 21.1 ± 0.3 years; body mass index (BMI): 20.9 ± 1.7 kg/m^2^; visual acuity as assessed using the Landolt “C” chart: 1.2 ± 0.4) were enrolled. The Research Ethics Committee of Shijoawate Gakuen University approved the study (approval code; 28-1), and all subjects provided written informed consent before participating in the study. This study was conducted in accordance with the Declaration of Helsinki.

### 2.2. Procedure and Apparatus

The subjects were instructed to stand upright on a force platform (AMTI, Watertown, MA, USA) covered with a foamed rubber layer (6-cm thick) with their arms alongside their body and their feet 5 cm apart. During 20-s long trials, COP displacement in ML and AP directions was recorded. The subjects were instructed to stand as still as possible with their eyes closed or gazing at a marker placed in front of them on a thin pole at eye-level height. Gazing markers were placed at distances of 0.5 m, 1.0 m, and 1.5 m in a random order. Marker sizes were adjusted to occupy approximately 2° of the participants’ central vision at each gazing distance (i.e., 1.7-cm diameter at 0.5 m, 3.4-cm diameter at 1.0 m, and 5.1-cm diameter at 1.5 m). The room was moderately illuminated, and the front wall was visible and covered with black cloth. COP was measured via the force platform at a sampling rate of 1000 Hz. The subjects completed two trials under each condition.

GVS (GVS intensity: 1 mA; duration: 7 s) was applied using a constant-current stimulator (SEN-3301, Nihon Khoden, Tokyo, Japan) at random, 10–13 s after the start of each trial. To induce a rightward body tilt, binaural bipolar stimulation was applied; electrodes (Ag/AgCl, 907 mm^2^) were placed over both mastoids, with the anode on the right [9]. To induce forward and backward body tilts, binaural monopolar stimulation was applied; to induce a forward body tilt, two anodes were placed on the forehead and cathodes on the right and left mastoids, and the opposite anode–cathode positions were used to induce a backward body tilt. The effect of GVS for leftward body tilt are reportedly the same as those of GVS for rightward body tilt [9]; thus, we only induced GVS for rightward body tilt.

### 2.3. Data Processing and Analysis

Before analysis, force plate data were low-pass filtered with a cut-off frequency of 20 Hz. We subsequently calculated the root mean square (RMS) value for the 7 s of COP data prior to and during GVS. Following this, we subtracted the pre-GVS RMS value from that of the GVS RMS value. This variable represents COP deviation during GVS, normalized to pre-GVS COP. Furthermore, this value also represents COP fluctuation, which was an additional interest in our study. The mean of the two trials under each condition was used for further analysis. Our data did not show a normal distribution, and thus we used Friedman’s test to compare the visual conditions in each direction (ML and AP) with the GVS configuration (rightward, forward, and backward tilting). Post-hoc comparisons were performed using Wilcoxon’s test. To evaluate the visual effect on spontaneous postural sway, we also compared the pre-GVS RMS in each visual condition using Wilcoxon’s test. Statistical significance was set at 5% using Holm’s correction method [11].

## 3. Results

In the ML direction of pre-GVS RMS comparison between visual conditions, values in the eyes-closed condition were significantly larger than values in the eye-open conditions (0.5 m, *p* = 0.002; 1.0 m, *p* = 0.002; 1.5 m, *p* = 0.004). The ML pre-GVS RMS value in the 1.0 m condition was significantly larger than that in the 0.5 m condition (*p* = 0.014). No significant differences were observed in the AP direction of pre-GVS RMS between visual conditions (Table 1).

**Figure 1:**
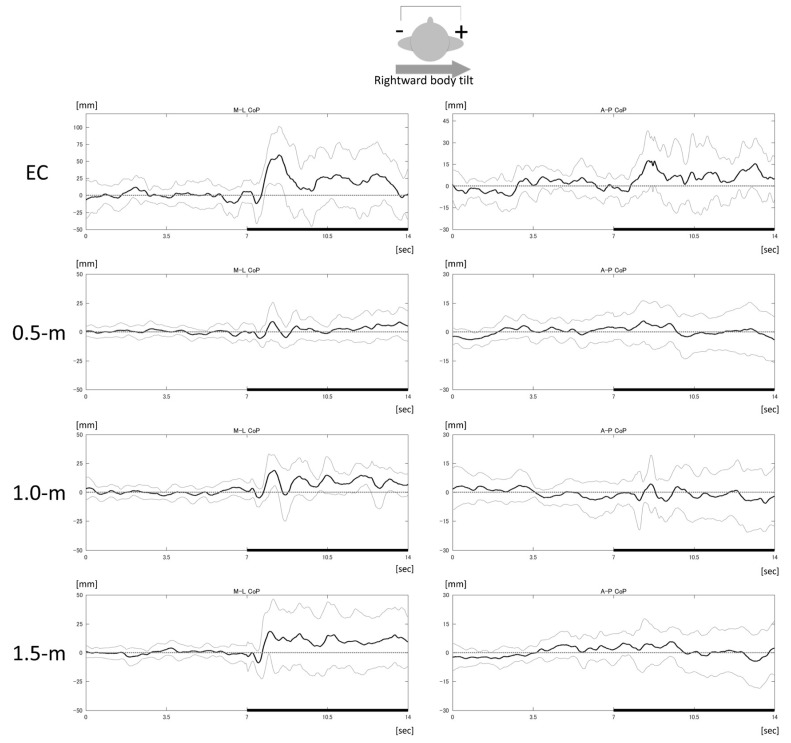
Mean and standard deviations of mediolateral (ML) (left) and anteroposterior (AP) (right) center of pressure (COP) in each visual condition, during galvanic vestibular stimulation (GVS)-induced rightward body tilting. The plotted data includes the mean (black line) and standard deviation (gray line) for 10 subjects. Positive values indicate rightward (left column) and forward (right column) COP displacements from the mean during the first 10 s of measurement (zero: gray dotted line). The duration of GVS application was 7–14 s (thick horizontal black line). EC: eyes closed; 0.5-m: gazing marker at 0.5 m in front of subject; 1.0-m: marker at 1.0 m; 1.5-m: marker at 1.5 m.

**Figure 2:**
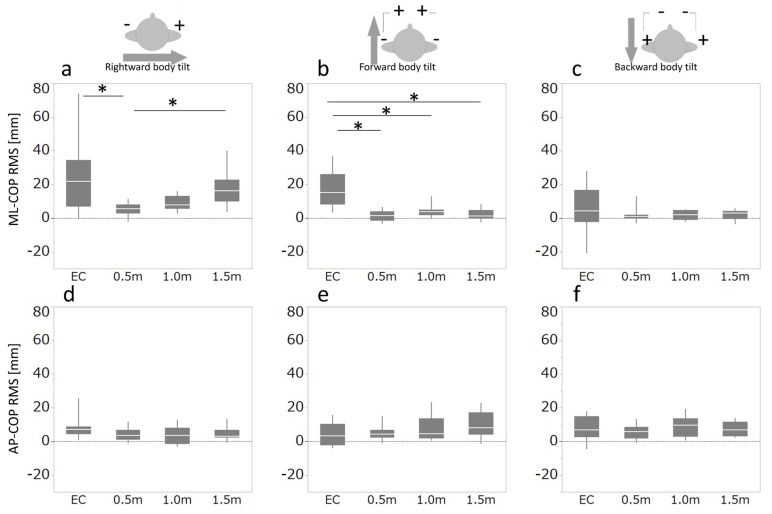
Root mean square (RMS) of anteroposterior (AP) and mediolateral (ML) center of pressure (COP) measurements during body-tilting galvanic vestibular stimulation (GVS). (**a**) ML COP RMS and (**d**) AP COP RMS during rightward body-tilting GVS. (**b**) ML COP RMS and (**e**) AP COP RMS during forward body-tilting GVS. (**c**) ML COP RMS and (**f**) AP COP RMS during backward body-tilting GVS. Box and whisker plots represent the interquartile maximum and minimum COP. The positive values correspond to the direction of body tilt induced by GVS. An asterisk denotes statistically significant differences between conditions (*p* < 0.05 with Holm’s correction). EC: eyes closed; 0.5-m: gazing marker at 0.5 m in front of subject; 1.0-m: marker at 1.0m; 1.5-m: marker at 1.5 m.

In the ML direction, significant visual condition-related differences were found during GVS with rightward (*χ*^2^ (3) = 12.6, *p* = 0.006, *V* = 0.65) (Figure 1) and forward (*χ*^2^ (3) = 14.0, *p* = 0.003, *V* = 0.69) body-tilting, but not during backward body-tilting (Figure 2a–c). During rightward body-tilting (Figure 2a), the ML COP RMS value for an eye-object distance of 0.5 m was smaller compared with the eyes-closed and 1.5 m ML COP RMS values (*Z* = 2.54, *p* = 0.006, *r* = 0.80; *Z* = 2.46, *p* = 0.01, *r* = 0.78, respectively). During GVS with forward body-tilting (Figure 2b), the ML COP RMS value in the eyes-closed condition was larger than those in the 0.5 m, 1.0 m, and 1.5 m conditions (*Z* = 2.75, *p* = 0.002, *r* = 0.87; *Z* = 2.65, *p* = 0.004, *r* = 0.84; *Z* = 2.65, *p* = 0.004, *r* = 0.84, respectively). In the AP direction, there were no significant differences in the COP RMS values for the rightward, forward, or backward body-tilting GVS configurations related to eye-object distances (Figure 2d–f).

## 4. Discussion

The present study examined the effect of eye-object distance on COP RMS during body-tilting GVS manipulations with healthy subjects. Our results showed that the eye-object distance effect was observed for body sway in the ML direction.

In previous studies, eye-object distance effects were evident for eye-object distances of up to 1 or 1.5 m [5]; the present results demonstrate that eye-object distance effects are also observed for smaller eye-object distances. In addition, this study demonstrated that eye-object distance effects are observed only in the ML direction during rightward body-tilting GVS manipulation. We thus propose that this effect can be attributed to the detection of oculomotor muscle proprioception and motion parallax, and these sensory inputs could assist in the regulation of ML body sway [3,8].

During forward body-tilting, there was a significant difference in ML COP RMS between eyes closed and the other conditions. However, there were no differences in ML COP RMS between the 0.5, 1.0, and 1.5 m marker distances. Forward body-tilting would not cause ML body sway alone; however, in the present study, participants stood on foamed rubber. We postulate that the observed ML body sway arose from the participants attempting to maintain balance against the forward manipulation while their eyes were closed. This behavior would explain the differences observed between the eyes-closed and eyes-open measurements.

No eye-object distance effect was observed during backward body-tilting GVS manipulations. It was reported that backward body-tilting GVS induces a slightly smaller COP deviation compared with forward body-tilting GVS [9]; thus, we speculated that the differences in ML COP induced by backward tilting were too small to detect any eye-object distance effects.

Small eye-object distances did not reduce the AP COP RMS during GVS (Figure 2d–f). For ML sway, baseline RMS (no applied GVS) showed less sway for small eye-object distances compared with that for further distances (Table 1). However, this effect was not observed for AP sway, which could imply that perceiving AP sway is more difficult than perceiving ML sway because the visual information is less available; AP sway causes oculomotor muscle proprioception by convergence [3], whereas for ML sway, oculomotor muscle proprioception results from lateral eye movement and motion parallax [5]. Thus, there was no eye-object distance effect on AP COP RMS, even during the body-tilting GVS. Furthermore, it was reported that COP deviations derived from GVS application in the AP direction are of approximately half the amplitude of those in the ML direction [12]. Considering the sample- and effect-size constraints of the present study, the absence of differences in AP COP deviation is consistent with previous findings.

Vision is often used for balance training in patients with impaired proprioceptive and/or vestibular function. Our findings in this study can offer information on how visual information works in rehabilitation settings. Small eye-object distance can effectively reduce body sway in the ML rather than the AP direction. This information might be useful for treating disorders such as pusher syndrome caused by a stroke, which can result in lateral body tilt.

A limitation of the present study concerns the use of peripheral vision. We measured eye-object distance effects under illuminated conditions; thus, participants could use their peripheral vison to reduce spontaneous body sway [4]. However, peripheral vison was kept constant across eye-object distances, so we conclude that it had a minimal effect on our results. In this study, we used 7 s of data to calculate experimental outcomes. Feedforward and feedback body control in response to visual information switches within 2 s after GVS onset [1]. Therefore, it is reasonable to analyze the first 2 s of data following GVS onset to separately detect feedforward and feedback effects. However, eye-object distance effects are supposedly combined with feedback and feedforward body control to maintain balance; we were interested in investigating these combined effects, and thus used 7 s of data for our analysis. Another limitation of this study concerns power; we obtained only two trials in each condition, whereas many studies have used at least 10 trials to suppress variances in GVS-evoked responses [9]. To address this concern, we have referred the standard error of the mean (SEM) of GVS responses from previous studies and compared the mean of those to our study. Generally, the results indicated that the mean was appropriate, and we believe that the number of trials in our study did not adversely affect the results. However, given that the current study is based on a low number of trials, we feel that this should be considered a limitation and is important to keep in mind when situating the findings within the broader literature. Likewise, we would encourage future replications of the study to further confirm the findings.

## 5. Conclusions

In conclusion, we examined the directionality of the eye-object distance effect using body-tilting GVS manipulations. Our results show that the eye-object distance effect is observed and regulated only in the ML direction of body sway. Furthermore, these effects are observed during rightward, but not forward or backward, body-tilting GVS.

## Figures and Tables

**Table 1:** Pre-galvanic vestibular stimulation (GVS) root mean square (RMS) comparison between each visual condition. ML—mediolateral; AP—anteroposterior.

	0.5 m	1.0 m	1.5 m	Eyes Closed
ML RMS [mm]	5.2 (4.2, 5.8)	6.7 (5.0, 8.0) *	6.4 (5.4, 7.4)	14.4 (11.6, 19.7) ^†,‡,§^
AP RMS [mm]	5.6 (4.5, 6.1)	6.8 (6.0, 7.1)	5.4 (4.4, 6.8)	6.8 (5.4, 9.0)

Values are represented as median (interquartile range). * = significance between 0.5 m and 1.0 m conditions, ^†^ = significance between the 0.5 m and eyes closed conditions, ^‡^ = significance between the 1.0 m and eye closed conditions, ^§^ = significance between the 1.5 m and eye closed conditions. Wilcoxon’s test was used for statistical analysis and significances were adjusted using Holm’s method.
